# Predicting axillary lymph node metastasis in breast cancer using a multimodal radiomics and deep learning model

**DOI:** 10.3389/fimmu.2024.1482020

**Published:** 2024-12-13

**Authors:** Fuyu Guo, Shiwei Sun, Xiaoqian Deng, Yue Wang, Wei Yao, Peng Yue, Shaoduo Wu, Junrong Yan, Xiaojun Zhang, Yangang Zhang

**Affiliations:** ^1^ Third Hospital of Shanxi Medical University, Shanxi Bethune Hospital, Shanxi Academy of Medical Sciences, Tongji Shanxi Hospital, Taiyuan, China; ^2^ Department of Urology, Peking Union Medical College Hospital, Chinese Academy of Medical Sciences and Peking Union Medical College, Beijing, China; ^3^ Department of Urology, Xinzhou People’s Hospital, Xinzhou, China; ^4^ Department of Urology, Datong Fifth People’s Hospital, Datong, China; ^5^ Department of Urology, Handan First Hospital, Handan, China; ^6^ Shanxi Bethune Hospital, Shanxi Academy of Medical Sciences, Tongji Shanxi Hospital, Third Hospital of Shanxi Medical University, Taiyuan, China; ^7^ Tongji Hospital, Tongji Medical College, Huazhong University of Science and Technology, Wuhan, China

**Keywords:** mammography, magnetic resonance imaging, machine learning, radiomics, deep learning, breast cancer, axillary lymph node metastasis

## Abstract

**Objective:**

To explore the value of combined radiomics and deep learning models using different machine learning algorithms based on mammography (MG) and magnetic resonance imaging (MRI) for predicting axillary lymph node metastasis (ALNM) in breast cancer (BC). The objective is to provide guidance for developing scientifically individualized treatment plans, assessing prognosis, and planning preoperative interventions.

**Methods:**

A retrospective analysis was conducted on clinical and imaging data from 270 patients with BC confirmed by surgical pathology at the Third Hospital of Shanxi Medical University between November 2022 and April 2024. Multiple sequence images from MG and MRI were selected, and regions of interest in the lesions were delineated. Radiomics and deep learning (3D-Resnet18) features were extracted and fused. The samples were randomly divided into training and test sets in a 7:3 ratio. Dimensionality reduction and feature selection were performed using the least absolute shrinkage and selection operator (LASSO) regression model, and other methods. Various machine learning algorithms were used to construct radiomics, deep learning, and combined models. These models were visualized and evaluated for performance using receiver operating characteristic curves, area under the curve (AUC), calibration curves, and decision curves.

**Results:**

The highest AUCs in the test set were achieved using radiomics-logistic regression (AUC = 0.759), deep learning-multilayer perceptron (MLP) (AUC = 0.712), and combined-MLP models (AUC = 0.846). The MLP model demonstrated strong classification performance, with the combined model (AUC = 0.846) outperforming both the radiomics (AUC = 0.756) and deep learning (AUC = 0.712) models.

**Conclusion:**

The multimodal radiomics and deep learning models developed in this study, incorporating various machine learning algorithms, offer significant value for the preoperative prediction of ALNM in BC.

## 1 Introduction

Breast cancer (BC) has surpassed lung cancer as the most common cancer among women worldwide and is the leading cause of cancer-related deaths in women globally (1). Prognosis and treatment strategies for BC vary depending on the molecular subtype, clinical stage, and histological grading (2). Axillary lymph node metastasis (ALNM) plays a critical role in BC clinical staging. Studies have shown that patients with BC and ALNM have a 14% lower 5-year survival rate compared to those without metastasis (3, 4). Preoperative adjuvant therapy or axillary lymph node dissection (ALND) can improve patient survival (5, 6). Early prediction of ALNM can help clinicians assess prognosis and develop individualized treatment plans for patients with BC. However, imaging assessments in clinical practice are often subjective, requiring fine-needle aspiration or postoperative histopathological biopsy for accurate results. Therefore, there is a need for accurate and noninvasive methods to evaluate ALNM.

Radiomics can be used to noninvasively extract high-dimensional data from images, revealing tumor-rated characteristics that are invisible to the naked eye. This approach can potentially provide more accurate interpretations of results, prognoses, and treatment predictions (7–9). Deep learning, a new field in artificial intelligence (AI) and machine learning, involves multilayer neural networks that mimic the architecture of the human brain. Convolutional neural networks, a highly effective deep learning method, are widely used in medical image analysis (10). BC imaging typically involves mammography (MG) and magnetic resonance imaging (MRI), each with strengths and weaknesses in distinguishing calcifications from soft tissues. Research indicates that combining both methods provides more comprehensive information (11, 12).

Previous studies have mostly relied on single MG or MRI images and individual machine-learning algorithms to construct radiomics or deep-learning models, which limits tumor information and hinders the identification of the most effective models. This study investigated the performance of multimodal radiomics combined with deep learning models, utilizing various machine learning algorithms based on MG and MRI, for the preoperative prediction of ALNM in patients with BC. The objective was to guide preoperative intervention, prognosis assessment, and the development of scientifically individualized treatment plans.

## 2 Materials and methods

### 2.1 Study cohort

A retrospective analysis was conducted on the clinical and radiological data of patients with BC confirmed by surgical pathology at the Third Hospital of Shanxi Medical University between November 2022 and April 2024. The inclusion criteria were as follows:

1. Underwent breast MRI and MG examinations with clear and complete images performed before biopsy, surgical treatment, or chemoradiotherapy.
2. An interval of ≤ 1 week between MG and MRI examinations.
3. BC confirmed through pathological results, with the axillary lymph node (ALN) status determined by ALND, fine-needle aspiration, or sentinel lymph node biopsy (SLNB), some of which were conducted prior to neoadjuvant therapy.

The exclusion criteria were as follows:

1. Primary breast tumor lesions that were too small lesions or had unclear boundaries, making it difficult to delineate the region of interest (ROI).
2. Occult, recurrent, or distant metastatic BC.
3. A history of other malignancies.
4. Receiving neoadjuvant therapy before the pathological evaluation of lymph nodes and imaging assessment.

**Figure 1:**
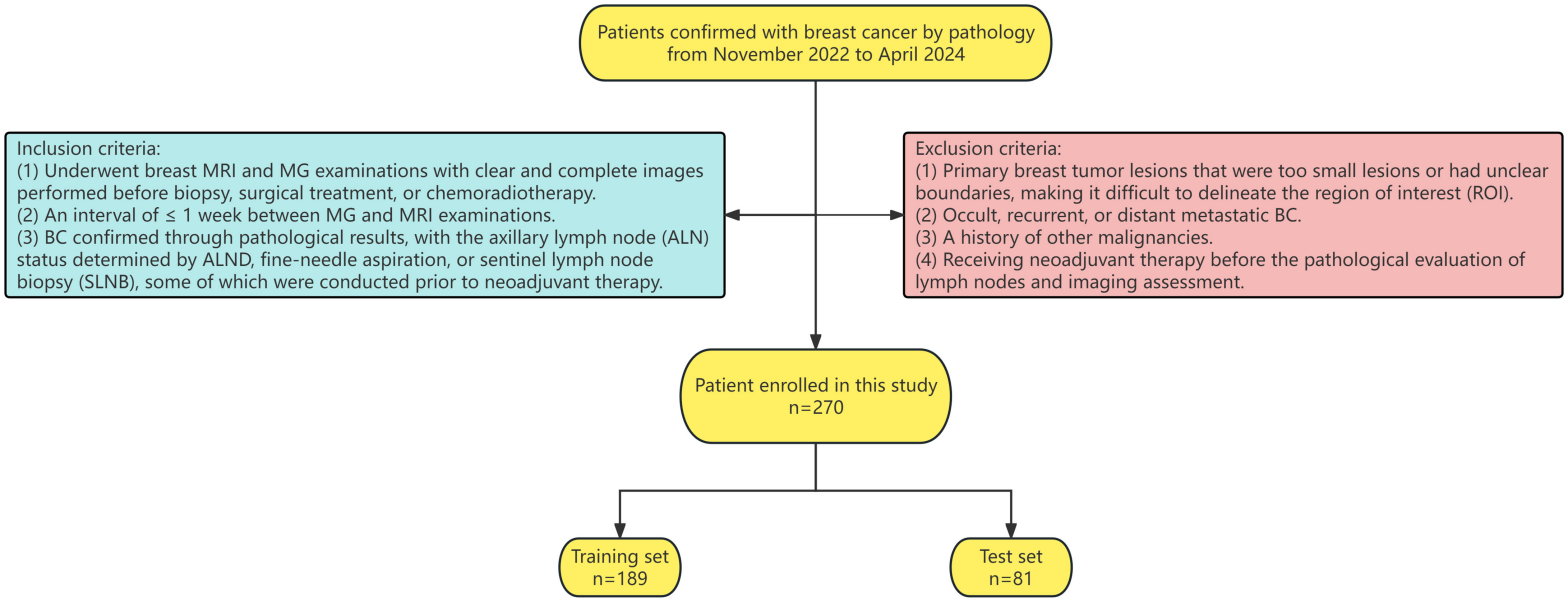
Flowchart of the patient enrolment.

The final analysis included 270 female patients. All samples were randomly divided into training and testing sets in a 7:3 ratio. Statistical analysis was conducted to verify that the baseline data were balanced between the groups. The ethics committee of the Third Hospital of Shanxi Medical University approved the study and waived the requirement for informed consent. The patient inclusion flowchart is presented in **Figure 1**.

### 2.2 Equipment and methods

#### 2.2.1 Equipment and image acquisition

MG was performed using the Hologic Selenia Dimensions digital mammography system for craniocaudal (CC) and mediolateral oblique (MLO) views of both breasts. The scanning sequence included the CC view of the affected side, the CC view unaffected side, the MLO view of the affected side, and the MLO view of the unaffected. Additional lateral views, spot compression views, and tomosynthesis were performed as needed.

MRI examinations were performed using a 3.0T MR scanner (Skyra, Siemens, Germany) equipped with an 18-channel dedicated coil. An apparent diffusion coefficient sequence was generated from two different b-value diffusion-weighted imaging (DWI) sequences. After mask scanning, Gd-DTPA (Gadopentetate Dimeglumine, Bayer, Germany) was injected into the antecubital vein at a rate of 2.5 mL/s using a power injector, at a dose of 0.2 mL/kg body weight, followed by a 20 mL saline flush for dynamic contrast-enhanced (DCE)-MRI. Enhanced images were continuously acquired over six phases after contrast injection. Detailed parameters are listed in **Supplementary Table 1**.

#### 2.2.2 Image preprocessing, segmentation, and feature extraction

For image preprocessing, the “N4BiasFieldCorrection” function from the “ANTsPy” package was first used to correct bias in MRI images, eliminating signal distortions caused by inhomogeneous magnetic fields and enhancing image uniformity and contrast. After that, the normalization function from the “nibabel” package was applied to standardize the MRI grayscale values, ensuring consistency across different images. All images were then resampled to a fixed resolution of 1×1×1 mm voxel size to standardize voxel spacing for spatial normalization.

After preprocessing, two experienced radiologists, blinded to the patients’ pathological results, used ITK-SNAP 4.2.0 software to delineate ROIs of the same lesions in the CC and MLO views of MG images as well as in T_2_-weighted imaging, DWI (b value = 800), apparent diffusion coefficient, and the second phase of DCE-MRI sequences. In cases with multiple lesions, the largest single lesion was selected. Radiologist A initially delineated the lesions for all patients, while Radiologist B randomly selected and delineated the lesions of 30 patients. Radiologist A repeated the delineation for these 30 patients 2 weeks later to test feature consistency. Any discrepancies were resolved by consensus.

Using the “Pyradiomics” package in Python 3.7, radiomics features were extracted from the delineated ROIs, including shape features, first-order statistics, texture features (second-order statistics) [Gray-Level Co-occurrence Matrix, Gray-Level Run Length Matrix, Gray-Level Dependence Matrix], higher-order statistics, frequency-domain features, filter-based features, and Local Binary Patterns features. Radiomics features from the different sequences were combined for subsequent screening. Deep learning features were extracted using a pretrained 3D ResNet model, with the input layer modified to accept a single-channel input and the final fully connected layer removed. The extracted deep-learning features were then combined across sequences. The combined model features were formed by integrating the deep learning features with the radiomic features that passed consistency testing. Radiomic, deep learning, and combined model features were independently analyzed in subsequent steps.

#### 2.2.3 Feature selection, model building, and evaluation

Radiomics features extracted from ROIs outlined by different doctors were assessed using a bidirectional consistency model to calculate the intraclass correlation coefficient (ICC), retaining features with an ICC > 0.9. Since deep-learning features are high-dimensional numerical vectors rather than manually defined, they did not require consistency testing.

A synthetic minority oversampling technique was used to balance the class distribution in the dataset by interpolating new minority class samples in the feature space (k = 5).

Z-score normalization was applied to the features to ensure that they were analyzed on the same scale. The mean and standard deviation of each feature were calculated from the training set, and these values were used to standardize both the training and validation sets to prevent data leakage.

After that, univariate analysis was performed to filter features. For normally distributed features with equal variance, independent sample t-tests were used; for normally distributed features with unequal variance, corrected t-test methods such as the Cochran & Cox, Satterthwaite, and Welch methods were applied; and for non-normally distributed features, the Mann-Whitney U test was employed. Features with p-values < 0.05 were retained.

Based on the univariate analysis results, Spearman correlation analysis was performed, and features with a correlation > 0.9 were randomly removed, repeating this process until all feature correlations were ≤ 0.9.

The Max-Relevance and min-redundancy method was applied to identify the features most relevant to the final output while minimizing redundancy among them.

Finally, LASSO regression was used for feature dimensionality reduction, with 20 repetitions of 10-fold cross-validation using different random seeds employed to select the feature subset corresponding to the optimal regularization parameter λ with the minimum mean squared error (MSE).

Using 20 repetitions of 5-fold cross-validation using different random seeds, the final selected radiomics and deep learning features were used to construct models with eight machine learning algorithms: logistic regression (LR), naive Bayes, support vector machine (SVM), k-nearest neighbors (KNN), extreme gradient boosting (XGBoost), light gradient boosting machine (LightGBM), adaptive boosting (AdaBoost), and multilayer perceptron (MLP).

**Figure 2:**
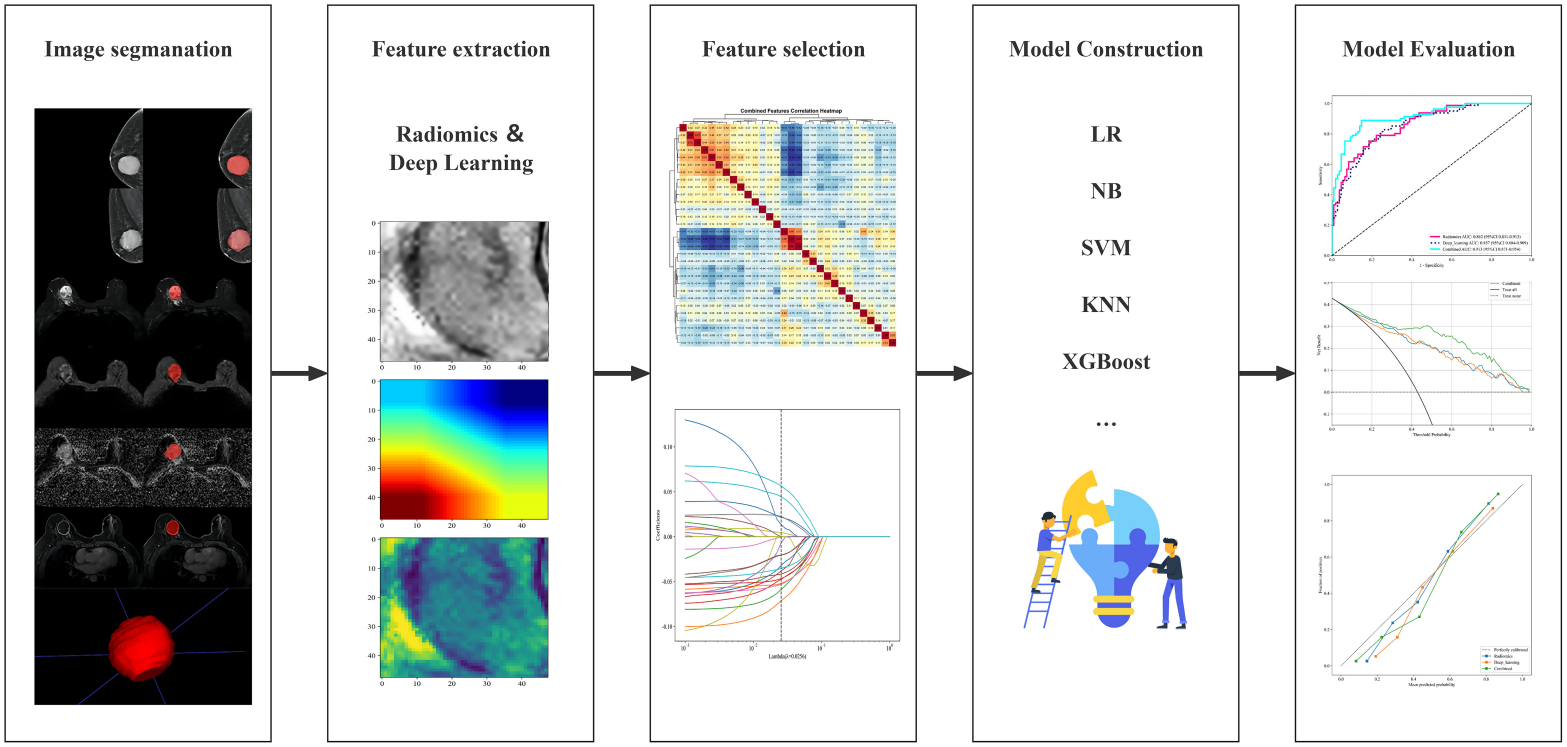
Flowchart of radiomics.

Receiver operating characteristic (ROC) curves were plotted for all the models, and metrics such as area under the curve (AUC), diagnostic accuracy, sensitivity, specificity, positive predictive value, negative predictive value, precision, recall, F1 score, and threshold were calculated. The best-performing algorithm model was selected, and waterfall plots were used to display the distribution of the model’s prediction probabilities. The DeLong test, net reclassification improvement index (NRI), and integrated discrimination improvement index (IDI) were used to compare model performance. Calibration curves (CAL) and decision curve analysis (DCA) were used to assess model fit and clinical benefits. The research workflow is illustrated in **Figure 2**.

#### 2.2.4 Statistical analysis

Statistical analyses were performed using R version 4.4.1. Continuous variables were presented as “mean ± standard deviation.” Independent-sample t-tests were used for normally distributed continuous variables; in contrast, the Mann–Whitney U test was used for non-normally distributed continuous variables. Categorical variables are expressed as frequencies and percentages, and comparisons were made using the chi-square test, continuity correction test, and Fisher’s exact test. Statistical significance was set at p < 0.05.

## 3 Results

### 3.1 Clinical characteristics

This study included 270 patients with BC: 117 (43.3%) with ALNM and 153 (56.7%) without ALNM. All patients were randomly divided into training (n = 189) and test (n = 81) sets in a 7:3 ratio. The training set comprised 80 (29.6%) patients with ALNM and 109 (40.4%) patients without ALNM; in contrast, the test set included 39 (14.4%) patients with ALNM and 42 (15.6%) patients without ALNM. Patient characteristics and comparisons between and within groups are presented in **Table 1**.

**Table 1:** The baseline clinical characteristics of patients.

	Training Set (N=189)		p.overall	Test Set (N=81)		p.overall
	ALNM (N=80)	Non-ALNM (N=109)		ALNM (N=39)	Non-ALNM (N=42)	
ALNM:			-			-
No	-	-		-	-	
Yes	-	-		-	-	
Age	51.0 (12.6)	53.3 (10.9)	0.197	53.2 (12.7)	55.7 (11.7)	0.372
BMI	24.8 (2.98)	24.8 (3.07)	0.936	24.4 (3.53)	24.8 (2.99)	0.545
Lesion Site:			0.987			0.928
Left	45 (56.2%)	60 (55.0%)		19 (48.7%)	19 (45.2%)	
Right	35 (43.8%)	49 (45.0%)		20 (51.3%)	23 (54.8%)	
MG BI-RADS:			<0.001			0.243
0	0 (0.00%)	1 (0.92%)		0 (0.00%)	0 (0.00%)	
1	0 (0.00%)	1 (0.92%)		0 (0.00%)	0 (0.00%)	
2	3 (3.75%)	8 (7.34%)		3 (7.69%)	2 (4.76%)	
3	2 (2.50%)	1 (0.92%)		0 (0.00%)	1 (2.38%)	
4a	1 (1.25%)	10 (9.17%)		0 (0.00%)	3 (7.14%)	
4b	3 (3.75%)	10 (9.17%)		2 (5.13%)	5 (11.9%)	
4c	31 (38.8%)	56 (51.4%)		16 (41.0%)	19 (45.2%)	
5	40 (50.0%)	22 (20.2%)		18 (46.2%)	12 (28.6%)	
MG ALNM:			<0.001			0.012
No	64 (80.0%)	107 (98.2%)		31 (79.5%)	41 (97.6%)	
Yes	16 (20.0%)	2 (1.83%)		8 (20.5%)	1 (2.38%)	
MG Spiculated Sign:			0.355			0.978
No	49 (61.3%)	75 (68.8%)		28 (71.8%)	29 (69.0%)	
Yes	31 (38.8%)	34 (31.2%)		11 (28.2%)	13 (31.0%)	
MG Lobulated Sign:			0.867			1.000
No	54 (67.5%)	76 (69.7%)		26 (66.7%)	29 (69.0%)	
Yes	26 (32.5%)	33 (30.3%)		13 (33.3%)	13 (31.0%)	
MG Calcification:			0.867			0.060
No	51 (63.7%)	67 (61.5%)		21 (53.8%)	32 (76.2%)	
Yes	29 (36.2%)	42 (38.5%)		18 (46.2%)	10 (23.8%)	
MRI BI-RADS:			0.018			0.007
3	2 (2.50%)	4 (3.67%)		0 (0.00%)	1 (2.38%)	
4a	1 (1.25%)	3 (2.75%)		0 (0.00%)	0 (0.00%)	
4b	1 (1.25%)	3 (2.75%)		1 (2.56%)	0 (0.00%)	
4c	9 (11.2%)	31 (28.4%)		4 (10.3%)	15 (35.7%)	
5	67 (83.8%)	68 (62.4%)		34 (87.2%)	26 (61.9%)	
MRI ALNM:			<0.001			0.009
No	39 (48.8%)	92 (84.4%)		21 (53.8%)	35 (83.3%)	
Yes	41 (51.2%)	17 (15.6%)		18 (46.2%)	7 (16.7%)	
MRI Spiculated Sign:			0.399			0.748
No	36 (45.0%)	57 (52.3%)		19 (48.7%)	23 (54.8%)	
Yes	44 (55.0%)	52 (47.7%)		20 (51.3%)	19 (45.2%)	
MRI Lobulated Sign:			0.893			0.978
No	61 (76.2%)	81 (74.3%)		28 (71.8%)	29 (69.0%)	
Yes	19 (23.8%)	28 (25.7%)		11 (28.2%)	13 (31.0%)	
MRI Tumor Margin:			0.640			0.947
Clear	57 (71.2%)	73 (67.0%)		30 (76.9%)	31 (73.8%)	
Indistinct	23 (28.7%)	36 (33.0%)		9 (23.1%)	11 (26.2%)	
DCIS:			0.022			0.117
No	80 (100%)	101 (92.7%)		39 (100%)	38 (90.5%)	
Yes	0 (0.00%)	8 (7.34%)		0 (0.00%)	4 (9.52%)	
IDC:			0.013			0.001
No	5 (6.25%)	22 (20.2%)		0 (0.00%)	10 (23.8%)	
Yes	75 (93.8%)	87 (79.8%)		39 (100%)	32 (76.2%)	
ER:			0.569			0.895
Negative	12 (15.0%)	21 (19.3%)		6 (15.4%)	5 (11.9%)	
Positive	68 (85.0%)	88 (80.7%)		33 (84.6%)	37 (88.1%)	
PR:			0.784			0.359
High expression	50 (62.5%)	66 (60.6%)		20 (51.3%)	28 (66.7%)	
Low expression	9 (11.2%)	10 (9.17%)		9 (23.1%)	6 (14.3%)	
Negative	21 (26.2%)	33 (30.3%)		10 (25.6%)	8 (19.0%)	
HER2:			0.460			0.452
Negative	57 (71.2%)	84 (77.1%)		31 (79.5%)	37 (88.1%)	
Positive	23 (28.7%)	25 (22.9%)		8 (20.5%)	5 (11.9%)	
Ki67:			0.003			0.074
High expression	69 (86.2%)	72 (66.1%)		35 (89.7%)	30 (71.4%)	
Low expression	11 (13.8%)	37 (33.9%)		4 (10.3%)	12 (28.6%)	
Molecular Subtype:			0.057			0.630
HER2-Enriched	6 (7.50%)	4 (3.67%)		3 (7.69%)	4 (9.52%)	
LuminalA	10 (12.5%)	29 (26.6%)		3 (7.69%)	7 (16.7%)	
LuminalB HER2-Negative	47 (58.8%)	46 (42.2%)		25 (64.1%)	20 (47.6%)	
LuminalB HER2-Positive	11 (13.8%)	19 (17.4%)		6 (15.4%)	8 (19.0%)	
TNBC	6 (7.50%)	11 (10.1%)		2 (5.13%)	3 (7.14%)	

MG/MRI ALNM, Suspicious ALNM in imaging reports; DCIS, Ductal carcinoma *in situ*; IDC, Invasive ductal carcinoma. Estrogen receptor (ER) and progesterone receptor (PR) are considered positive if ≥1% of cells show nuclear staining. PR expression is classified as low if ≤20% and high if >20%. HER2 (Human epidermal growth factor receptor 2) is deemed positive if the result is 3+ or if FISH indicates gene amplification; it is considered negative if the result is 0+, 1+, or if FISH indicates no amplification. Ki67 is classified as low expression if ≤14% of cells show nuclear staining and high expression if >14%.

### 3.2 Feature selection and machine learning model construction

**Figure 3:**
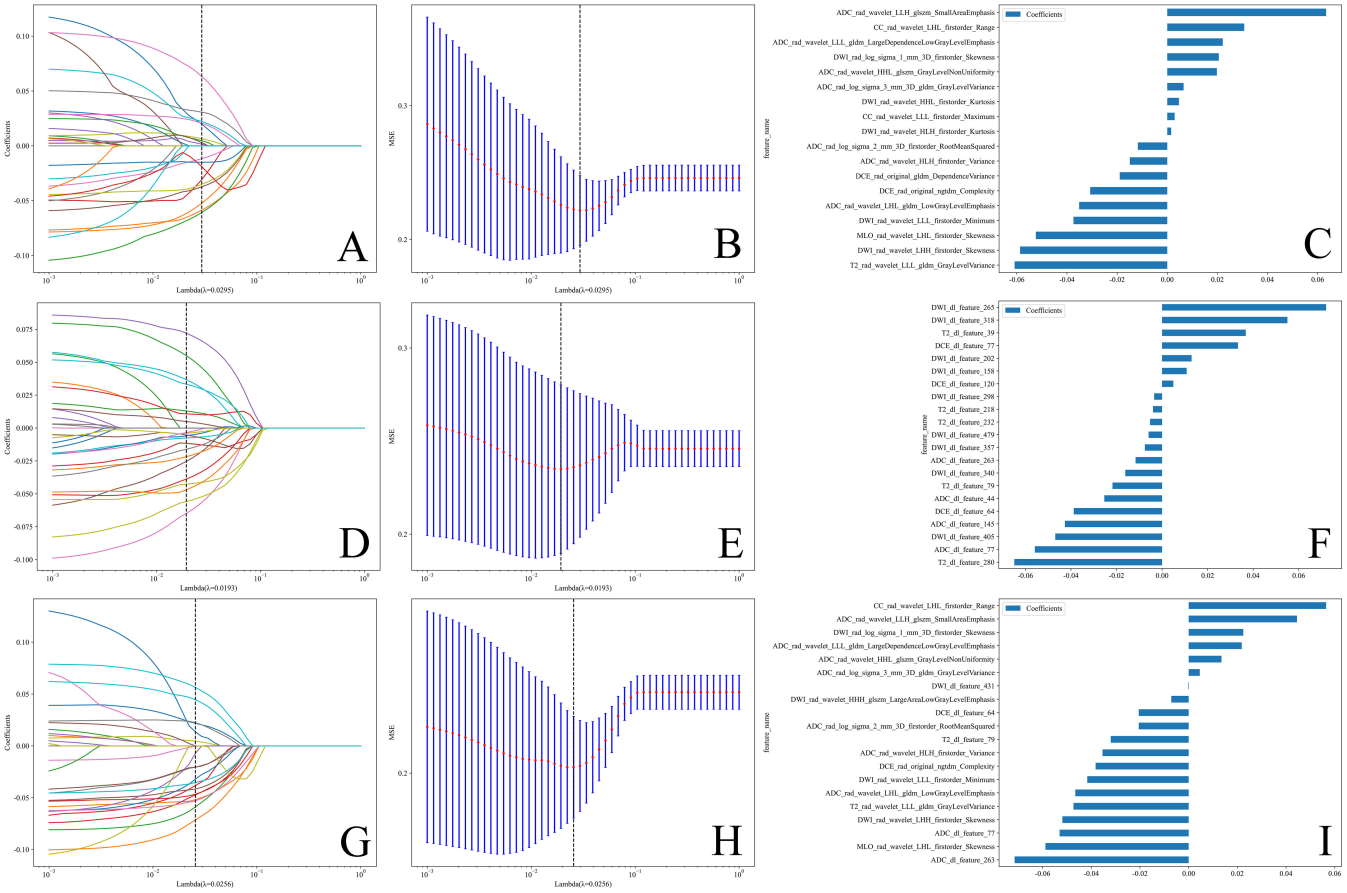
**(A, D, G)** Coefficients of LASSO 10 fold cross validation. **(B, E, H)** MSE of LASSO 10 fold cross validation.The feature subset corresponding to the λ value with the minimum MSE was retained. **(C, F, I)** Weights of features in the final subset. **(A–C)** Radiomics features. **(D–F)** Deep learning features. **(G–I)** Combined features.

**Table 2** lists the number of features retained at each step of the feature selection process and the corresponding figure numbers for visualizations. The feature selection process using LASSO regression is shown in **Figure 3**, while the final selected features and their coefficients are listed in **Supplementary Table 2**.

**Table 2:** Feature Counts.

	MG+MRI	ICC	Univariate Analysis	Spearman Correlation Analysis	mRMR	LASSO 10-Fold Cross-Validation
Corresponding Figures	-	**Supplementary Figure 1**	**Supplementary Figure 2**	-	**Supplementary Figure 3**	**Figure 3** and **Supplementary Figure 4**
Radiomics Features	6222	4291	845	190	30	18
Deep Learning Features	3072	-	123	97	30	21
Combined Features	-	7363	433	291	30	20

### 3.3 Model performance evaluation and visualization

**Figure 4:**
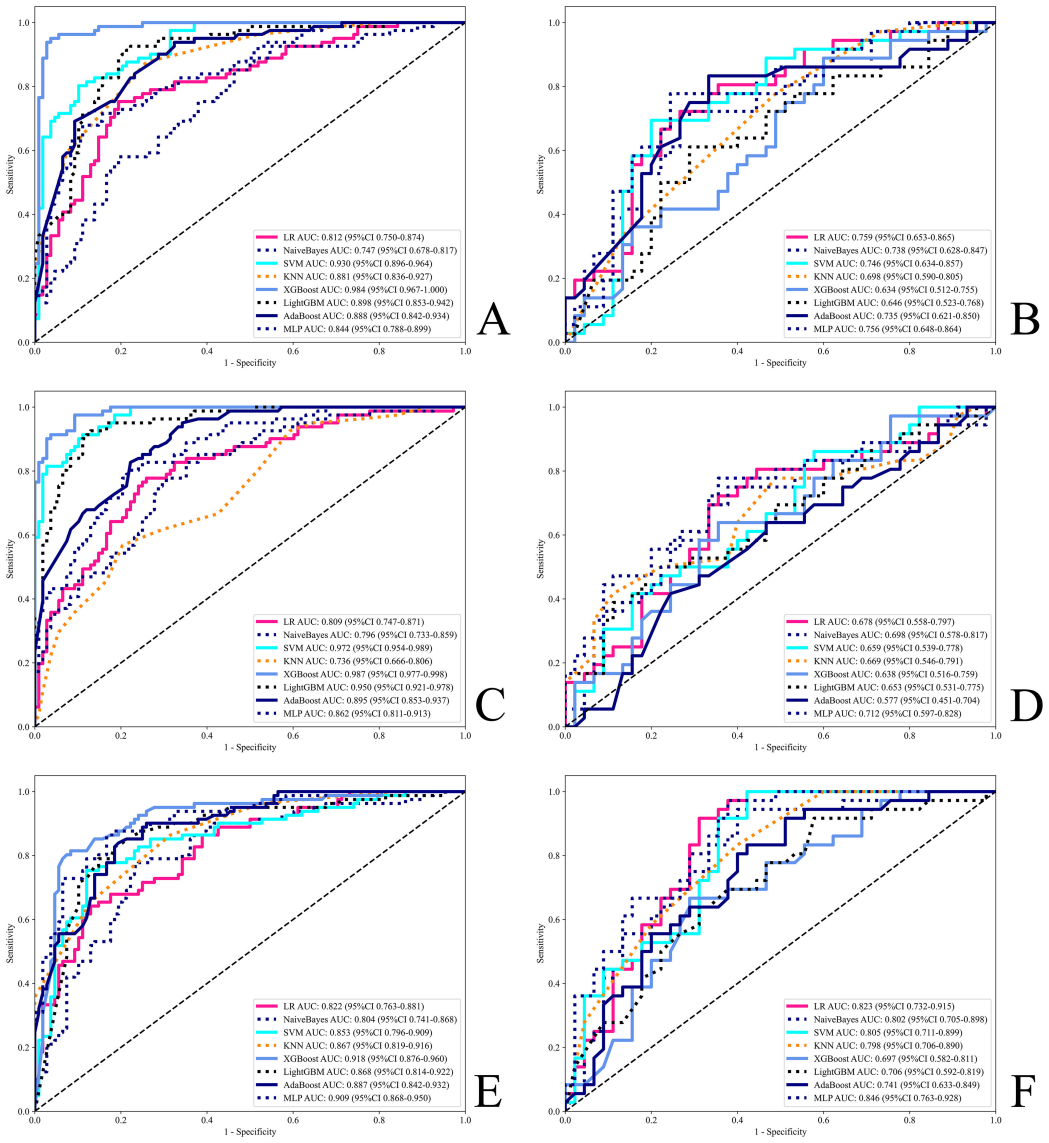
ROC for all models. **(A, B)** Radiomics models. **(C, D)** Deep learning models. **(E, F)** Combined models. **(A, C, E)** Training set. **(B, D, F)** Test set.

The diagnostic performance is presented in **Supplementary Table 3**, and the ROC curves are shown in **Figure 4**, indicating that all models performed well in both the test and training cohorts. Among all machine learning algorithms, the AUCs of the combined models were improved compared to those of the radiomics or deep learning models alone. The classifers with the highest perfomance in the test set for the three feature subset models were as follows:

Accuracy: Radiomics-MLP (0.753), Deep Learning-MLP (0.691), Combined-LR (0.778);

AUC: Radiomics-LR (0.759), Deep Learning-MLP (0.712), and Combined-MLP (0.846);

F1 score: Radiomics-MLP (0.730), Deep Learning-MLP (0.684), Combined-MLP (0.782);

Sensitivity: Radiomics-XGBoost (0.861), Deep Learning-SVM (0.833), Combined-MLP (0.944);

Specificity: Radiomics-KNN (0.867), Deep Learning-LightGBM (0.889), Combined-KNN (0.800);

PPV: Radiomics-SVM (0.727), Deep Learning-KNN (0.800), Combined-KNN (0.700);

NPV: Radiomics-AdaBoost (0.811), Deep Learning-MLP (0.763), Combined-SVM (0.963).

The MLP model demonstrated superior overall performance across the three different feature subset models, as detailed below:

Radiomics-MLP: AUC = 0.756, Accuracy = 0.753, F1 Score = 0.730;

Deep Learning-MLP: AUC = 0.712, Accuracy = 0.691, F1 Score = 0.684;

Combined-MLP: AUC = 0.846, Accuracy = 0.765, F1 Score = 0.782.

Therefore, this algorithm was selected for further interpretation of the study results.

The ROC, DCA, and CAL of the MLP model are shown in **Supplementary Figure 5**, the waterfall plot in **Supplementary Figure 6**, and the DeLong test, NRI, and IDI plots in **Supplementary Figure 7**. The test set results indicate that the combined model showed a statistically significant difference over single-modality models according to the DeLong test (P=0.043 for Combined-MLP vs. Radiomics-MLP; P=0.038 for Combined-MLP vs. Deep Learning-MLP). Additionally, NRI and IDI metrics showed improvements (NRI=0.074 and IDI=0.126 for Combined-MLP vs. Radiomics-MLP; NRI=0.213 and IDI=0.147 for Combined-MLP vs. Deep Learning-MLP). The ROC curve of the MLP model demonstrated excellent classification performance, with the AUC of the combined model in the test set (AUC = 0.846) showing an improvement compared with the radiomics (AUC = 0.756) and deep learning models (AUC = 0.712).

## 4 Discussion

The ALN is the most common site of metastasis in patients with BC. Determining ALNM is crucial for clinical staging and prognosis assessment because it helps in planning surgical and adjuvant treatment strategies (13). For example, depending on the metastasis, patients may need to receive neoadjuvant chemotherapy to reduce the size of the tumors and metastatic lymph nodes, increasing the success rate of surgery and the possibility of breast-conserving surgery. It can also assist radiologists in precisely targeting the radiation therapy area, thereby improving treatment efficacy and minimizing damage to normal tissues (14, 15). Imaging assessments of ALNM rely heavily on the subjective experience of physicians. SLNB and ALND can improve patient survival. However, these two methods have drawbacks. SLNB shows a high negative rate of 70–80%, indicating unnecessary procedures (16). ALND can lead to complications such as arm numbness and upper limb lymphedema (17). Therefore, we aimed to predict ALNM preoperatively to guide clinicians in preoperative intervention, prognostic assessment, and formulation of scientifically individualized treatment plans for BC.

In recent years, the field of AI has experienced unprecedented growth, significantly affecting various domains, including healthcare. Among the numerous applications, AI has shown remarkable potential for the early detection of cancer, prediction of treatment response, and prognosis assessment through advanced techniques, such as radiomics and deep learning (9, 10). This new technology offers accurate and noninvasive methods for predicting the pathological indicators of BC mentioned above. MG is a commonly used screening method for BC, with a sensitivity of approximately 75%, and is capable of detecting microcalcifications. However, its sensitivity decreases in women with dense breast tissue. In two-dimensional imaging, the overlap of normal fibroglandular tissue can mimic malignant features, leading to a higher false positive rate (18). MRI is suitable for screening high-risk patients with BC, with a sensitivity as high as 92%, and provides more detailed anatomical information that helps assess the extent and invasiveness of tumors. However, calcifications may not produce strong signals, making it difficult to distinguish them from the surrounding tissues. Additionally, MRI is sensitive to patient movement and metal implants during imaging, which can lead to artifacts that affect the image quality (19). While radiologists are highly skilled in interpreting medical images, their assessments are inherently subjective and can be influenced by factors such as experience, fatigue, and cognitive bias. This subjectivity can sometimes lead to variability in diagnosis and assessment. In contrast, radiomics and deep learning models offer a more objective and standardized approach to image analysis (20). Numerous studies have demonstrated that radiomics or deep learning models based on MG or MRI perform well in predicting BC risk, ALNM, histological grading, and response to neoadjuvant therapy, as well as in differentiating between benign and malignant breast masses and molecular subtypes of BC (21–27). Recent studies by Yuchen and Ma et al. found that radiomics models constructed by combining MG and MRI outperformed those based on a single sequence (11, 12). Additionally, research by Manon and Xue et al. demonstrated that models combining radiomics with deep learning outperform models using either method alone (28, 29). Inspired by the study mentioned above, we combined MG with MRI, radiomics, and deep learning to construct models using various machine-learning algorithms.

ResNet, proposed by He et al. in 2015, addresses the common issues of vanishing and exploding gradients in deep neural network training (30). This network architecture introduces a residual learning framework that enables the training of considerably deeper networks (31, 32). We utilize ResNet-18, a shallower version in the ResNet family. It has fewer parameters and lower computational complexity but still provides efficient feature extraction capabilities. It is widely used in various computer vision tasks.

Previous studies have established radiomics models to predict ALNM by delineating ROIs in the ALN region (3, 33). Our study demonstrated that models predicting ALNM based on ROIs drawn around breast tumors exhibit strong performance. This suggests that the imaging features of the primary tumor not only reflect the internal structure of the tumor but may also reveal insights into the tumor’s biological behavior, aggressiveness, and interactions with lymph nodes.

In the final selected feature subset, deep learning features did not include those from the MG sequence because MG sequence features were excluded owing to their high correlation with features from other sequences. Several combined features overlapped with the selected radiomics and deep learning features. We also attempted other methods to construct combined models; however, their performances did not match the approach used in this study. The specific methods and possible reasons are as follows: when combining independently selected radiomics and deep learning features, the interactions between features were not considered, leading to the exclusion of weak features that could play an important role in the combined feature set, ultimately resulting in a combined feature set that fails to capture the complementary information between the two types of features. Using the predicted probability values from radiomics and deep learning models as new features or taking their mean while simplifying the model complexity may result in information loss. This is because probability values typically reflect a model’s overall confidence in a specific sample rather than fully representing the complex relationships among all potential features (34, 35).

When comparing baseline data between the ALNM and non-ALNM groups, we found significant statistical differences in features such as MG/MRI ALNM and MG/MRI BI-RADS. However, after further selection of these clinical features and combining them with radiomics or deep learning models, the diagnostic performance in the test set did not show significant improvement and even decreased. We speculate that this may be due to the high correlation between clinical features and radiomics or deep learning features, leading to information redundancy when combined, which did not provide additional useful information to the model but instead increased its complexity. Additionally, our relatively small sample size may not represent the characteristics of the entire patient population, leading to a higher risk of overfitting (36, 37).

The MLP is a type of feedforward artificial neural network, composed of multiple layers of perceptrons, capable of handling highly nonlinear and complex problems through multiple hidden layers and nonlinear activation functions. Rosenblatt first proposed the perceptron model in the 1960s, and MLP is a multilayer extension. In this study, the combined model built with the MLP algorithm (AUC=0.846, Accuracy=0.765) outperformed the radiomics model (AUC=0.756, Accuracy=0.753) and the deep learning model (AUC=0.712, Accuracy=0.691). It was also more accurate than subjective assessments by radiologists (Accuracy for MG=0.637, Accuracy for MRI=0.689) and exceeded the performance of the model constructed by Hua, Y. et al., which combined MG and MRI but extracted only radiomics features (AUC=0.793, Accuracy=0.750) (11). The DCA indicates that all MLP models have higher net benefits than traditional decision strategies. CAL showed a good fit between the model’s predicted probabilities and actual outcomes. The waterfall plot visually illustrates the changes in individual samples within the model predictions. Most samples exhibited significant classification effects, with changes in the predicted probabilities showing a clear gradient, further validating the strong predictive ability of the model at the individual level. The waterfall plot also highlights a few misclassified samples, indicating the areas in which the model requires improvement. The DeLong test showed a significant statistical difference, and both the NRI and IDI were not vanishing, indicating that the MLP model can effectively integrate radiomics and deep learning features (38).

This study has a few limitations:

1. It was a single-center study with a small sample size and lacked external validation from other institutions, which may introduce selection bias.
2. Only the second-phase features of DCE-MRI were extracted; future research could include other phases or ultrasound images.

## 5 Conclusion

Multimodal radiomics, deep learning, and combined models based on MG and MRI constructed using various machine learning algorithms demonstrated good performance in the preoperative prediction of ALNM. These models may provide valuable guidance for physicians in preoperative intervention, prognostic assessment, and the development of scientifically individualized treatment plans for BC.

## Data Availability

The original contributions presented in the study are included in the article/**Supplementary Materials**, further inquiries can be directed to the corresponding authors.
