# Supplementary material for: Predicting axillary lymph node metastasis in breast cancer using a multimodal radiomics and deep learning model
Source: Front Immunol. 2024 Dec 13;15:1482020. doi: 10.3389/fimmu.2024.1482020 (PMC11671510; doi:10.3389/fimmu.2024.1482020)
Supplement: Supplementary file 1 [file DataSheet1.docx]

**Supplementary Material**

Supplementary Table 1. MRI sequences and scanning parameters. TR: Repetition time. TE: Echo time. T_1_WI: T_1_-weighted imaging. T_2_WI: T_2_-weighted imaging. DWI: diffusion-weighted imaging. DCE: dynamic contrast-enhanced.

Supplementary Table 2. Features and Coefficients from LASSO Regression. These features will be used to construct models using various machine learning algorithms. rad: Radiomics features. dl: Deep learning features.

Supplementary Table 3. Diagnostic performance for all models.

Supplementary Fig. 1. ICC index plot. Each point represents a radiomics feature. We retained the radiomics features with an ICC > 0.9 (above the red line).

Supplementary Fig. 2. Heatmaps of feature clustering analysis. Features selected through univariate analysis were used for plotting. Red indicates high feature values, while blue indicates low values.

Supplementary Fig. 3. Heatmaps of feature correlations after Spearman correlation test and mRMR, showing all feature correlations < 0.9.

Supplementary Fig. 4. Repeated 10-Fold Cross-Validation with Different Random Seeds. A: Radiomics models. B: Deep learning models. C: Combined models.

Supplementary Fig. 5. ROC, CAL, and DCA of the MLP model. A, B: ROC. C, D: CAL. E, F: DCA. A, C, E: Training set. B, D, F: Test set.

Supplementary Fig. 6. Waterfall plot of the MLP model. Blue columns (Label-0): Non-TNBC patients. Yellow columns (Label-1): TNBC patients. A, B: Radiomics models. C, D: Deep learning models. E, F: Combined models. A, C, E: Training set. B, D, F: Test set.

Supplementary Fig. 7. The results of the DeLong test, NRI, and IDI for the MLP model.

| Sequence | TR | TE | Field of view | Matrix | Slice thickness | Flip angle | b value |
| --- | --- | --- | --- | --- | --- | --- | --- |
|  | (ms) | (ms) | (mm) |  | (mm) | (°) | (s/mm2) |
| T_1_WI | 5.43 | 2.46 | 340×340 | 512×512 | 4 | 20 | - |
| T_2_WI | 4160 | 69 | 340×340 | 384×384 | 4 | 80 | - |
| DWI | 8320 | 60 | 207×340 | 180×110 | 4 | 180 | 0/800 |
| DCE | 4.45 | 1.68 | 360×360 | 448×448 | 1.6 | 10 | - |

Supplementary Table 1. MRI sequences and scanning parameters. TR: Repetition time. TE: Echo time. T_1_WI: T_1_-weighted imaging. T_2_WI: T_2_-weighted imaging. DWI: diffusion-weighted imaging. DCE: dynamic contrast-enhanced.

| **Radiomics Features** | |
| --- | --- |
| Constant | 0.428571429 |
| ADC_rad_log_sigma_2_mm_3D_firstorder_RootMeanSquared | -0.011728 |
| ADC_rad_log_sigma_3_mm_3D_gldm_GrayLevelVariance | 0.006513 |
| ADC_rad_wavelet_HHL_glszm_GrayLevelNonUniformity | 0.01978 |
| ADC_rad_wavelet_HLH_firstorder_Variance | -0.014928 |
| ADC_rad_wavelet_LHL_gldm_LowGrayLevelEmphasis | -0.035142 |
| ADC_rad_wavelet_LLH_glszm_SmallAreaEmphasis | 0.063365 |
| ADC_rad_wavelet_LLL_gldm_LargeDependenceLowGrayLevelEmphasis | 0.022148 |
| CC_rad_wavelet_LHL_firstorder_Range | 0.030691 |
| CC_rad_wavelet_LLL_firstorder_Maximum | 0.002947 |
| DCE_rad_original_gldm_DependenceVariance | -0.018954 |
| DCE_rad_original_ngtdm_Complexity | -0.030727 |
| DWI_rad_log_sigma_1_mm_3D_firstorder_Skewness | 0.020561 |
| DWI_rad_wavelet_HHL_firstorder_Kurtosis | 0.004646 |
| DWI_rad_wavelet_HLH_firstorder_Kurtosis | 0.001549 |
| DWI_rad_wavelet_LHH_firstorder_Skewness | -0.0587 |
| DWI_rad_wavelet_LLL_firstorder_Minimum | -0.037397 |
| MLO_rad_wavelet_LHL_firstorder_Skewness | -0.052421 |
| T2_rad_wavelet_LLL_gldm_GrayLevelVariance | -0.060884 |
| **Deep Learning Features** | |
| Constant | 0.428571429 |
| ADC_dl_feature_145 | -0.042652 |
| ADC_dl_feature_263 | -0.011636 |
| ADC_dl_feature_44 | -0.025361 |
| ADC_dl_feature_77 | -0.055847 |
| DCE_dl_feature_120 | 0.00495 |
| DCE_dl_feature_64 | -0.038773 |
| DCE_dl_feature_77 | 0.033336 |
| DWI_dl_feature_158 | 0.010786 |
| DWI_dl_feature_202 | 0.012957 |
| DWI_dl_feature_265 | 0.072019 |
| DWI_dl_feature_298 | -0.003468 |
| DWI_dl_feature_318 | 0.055034 |
| DWI_dl_feature_340 | -0.016138 |
| DWI_dl_feature_357 | -0.007545 |
| DWI_dl_feature_405 | -0.046905 |
| DWI_dl_feature_479 | -0.005904 |
| T2_dl_feature_218 | -0.003984 |
| T2_dl_feature_232 | -0.005263 |
| T2_dl_feature_280 | -0.064869 |
| T2_dl_feature_39 | 0.036756 |
| T2_dl_feature_79 | -0.021762 |
| **Combined Features** | |
| Constant | 0.428571429 |
| ADC_dl_feature_263 | -0.071502 |
| ADC_dl_feature_77 | -0.053028 |
| ADC_rad_log_sigma_2_mm_3D_firstorder_RootMeanSquared | -0.020509 |
| ADC_rad_log_sigma_3_mm_3D_gldm_GrayLevelVariance | 0.004557 |
| ADC_rad_wavelet_HHL_glszm_GrayLevelNonUniformity | 0.013471 |
| ADC_rad_wavelet_HLH_firstorder_Variance | -0.03538 |
| ADC_rad_wavelet_LHL_gldm_LowGrayLevelEmphasis | -0.046583 |
| ADC_rad_wavelet_LLH_glszm_SmallAreaEmphasis | 0.044487 |
| ADC_rad_wavelet_LLL_gldm_LargeDependenceLowGrayLevelEmphasis | 0.021789 |
| CC_rad_wavelet_LHL_firstorder_Range | 0.056452 |
| DCE_dl_feature_64 | -0.020506 |
| DCE_rad_original_ngtdm_Complexity | -0.038232 |
| DWI_dl_feature_431 | -0.000217 |
| DWI_rad_log_sigma_1_mm_3D_firstorder_Skewness | 0.022432 |
| DWI_rad_wavelet_HHH_glszm_LargeAreaLowGrayLevelEmphasis | -0.007142 |
| DWI_rad_wavelet_LHH_firstorder_Skewness | -0.051879 |
| DWI_rad_wavelet_LLL_firstorder_Minimum | -0.041659 |
| MLO_rad_wavelet_LHL_firstorder_Skewness | -0.05884 |
| T2_dl_feature_79 | -0.032004 |
| T2_rad_wavelet_LLL_gldm_GrayLevelVariance | -0.047315 |

Supplementary Table 2. Features and Coefficients from LASSO Regression. These features will be used to construct models using various machine learning algorithms. rad: Radiomics features. dl: Deep learning features.

| Model | Cohort | Accuracy | AUC（95% CI） | Sensitivity | Specificity | PPV | NPV | F1 Score | Threshold |
| --- | --- | --- | --- | --- | --- | --- | --- | --- | --- |
| Radiomics-LR | training | 0.778 | 0.812（0.750 - 0.874） | 0.741 | 0.806 | 0.741 | 0.806 | 0.741 | 0.568 |
| Radiomics-LR | test | 0.716 | 0.759（0.653 - 0.865） | 0.694 | 0.733 | 0.676 | 0.750 | 0.685 | 0.558 |
| Radiomics-NaiveBayes | training | 0.704 | 0.747（0.678 - 0.817） | 0.568 | 0.806 | 0.687 | 0.713 | 0.622 | 0.535 |
| Radiomics-NaiveBayes | test | 0.704 | 0.738（0.628 - 0.847） | 0.694 | 0.711 | 0.658 | 0.744 | 0.676 | 0.317 |
| Radiomics-SVM | training | 0.852 | 0.930（0.896 - 0.964） | 0.790 | 0.898 | 0.853 | 0.851 | 0.821 | 0.556 |
| Radiomics-SVM | test | 0.741 | 0.746（0.634 - 0.857） | 0.667 | 0.800 | 0.727 | 0.750 | 0.696 | 0.468 |
| Radiomics-KNN | training | 0.783 | 0.881（0.836 - 0.927） | 0.593 | 0.926 | 0.857 | 0.752 | 0.701 | 0.600 |
| Radiomics-KNN | test | 0.630 | 0.698（0.590 - 0.805） | 0.333 | 0.867 | 0.667 | 0.619 | 0.444 | 0.600 |
| Radiomics-XGBoost | training | 0.952 | 0.984（0.967 - 1.000） | 0.938 | 0.963 | 0.950 | 0.954 | 0.944 | 0.582 |
| Radiomics-XGBoost | test | 0.605 | 0.634（0.512 - 0.756） | 0.861 | 0.400 | 0.534 | 0.783 | 0.660 | 0.301 |
| Radiomics-LightGBM | training | 0.836 | 0.898（0.853 - 0.942） | 0.914 | 0.778 | 0.755 | 0.923 | 0.827 | 0.459 |
| Radiomics-LightGBM | test | 0.654 | 0.646（0.523 - 0.768） | 0.583 | 0.711 | 0.618 | 0.681 | 0.600 | 0.508 |
| Radiomics-AdaBoost | training | 0.788 | 0.888（0.842 - 0.934） | 0.889 | 0.713 | 0.699 | 0.895 | 0.783 | 0.491 |
| Radiomics-AdaBoost | test | 0.728 | 0.735（0.621 - 0.850） | 0.806 | 0.667 | 0.659 | 0.811 | 0.725 | 0.484 |
| Radiomics-MLP | training | 0.794 | 0.844（0.788 - 0.899） | 0.667 | 0.889 | 0.818 | 0.780 | 0.735 | 0.567 |
| Radiomics-MLP | test | 0.753 | 0.756（0.648 - 0.864） | 0.750 | 0.756 | 0.711 | 0.791 | 0.730 | 0.544 |
| Deep Learning-LR | training | 0.751 | 0.809（0.747 - 0.871） | 0.765 | 0.741 | 0.689 | 0.808 | 0.725 | 0.461 |
| Deep Learning-LR | test | 0.667 | 0.678（0.558 - 0.797） | 0.694 | 0.644 | 0.610 | 0.725 | 0.649 | 0.364 |
| Deep Learning-NaiveBayes | training | 0.720 | 0.796（0.733 - 0.859） | 0.815 | 0.648 | 0.635 | 0.824 | 0.714 | 0.184 |
| Deep Learning-NaiveBayes | test | 0.667 | 0.698（0.578 - 0.817） | 0.722 | 0.622 | 0.605 | 0.737 | 0.658 | 0.215 |
| Deep Learning-SVM | training | 0.894 | 0.972（0.954 - 0.990） | 0.901 | 0.889 | 0.859 | 0.923 | 0.880 | 0.437 |
| Deep Learning-SVM | test | 0.605 | 0.659（0.539 - 0.778） | 0.833 | 0.422 | 0.536 | 0.760 | 0.652 | 0.310 |
| Deep Learning-KNN | training | 0.672 | 0.736（0.666 - 0.806） | 0.457 | 0.833 | 0.673 | 0.672 | 0.544 | 0.650 |
| Deep Learning-KNN | test | 0.667 | 0.669（0.546 - 0.791） | 0.333 | 0.933 | 0.800 | 0.636 | 0.471 | 0.750 |
| Deep Learning-XGBoost | training | 0.931 | 0.987（0.977 - 0.998） | 0.963 | 0.907 | 0.886 | 0.970 | 0.923 | 0.439 |
| Deep Learning-XGBoost | test | 0.630 | 0.638（0.516 - 0.759） | 0.611 | 0.644 | 0.579 | 0.674 | 0.595 | 0.477 |
| Deep Learning-LightGBM | training | 0.889 | 0.950（0.921 - 0.978） | 0.914 | 0.870 | 0.841 | 0.931 | 0.876 | 0.479 |
| Deep Learning-LightGBM | test | 0.654 | 0.653（0.531 - 0.775） | 0.361 | 0.889 | 0.722 | 0.635 | 0.481 | 0.600 |
| Deep Learning-AdaBoost | training | 0.778 | 0.895（0.853 - 0.937） | 0.938 | 0.657 | 0.673 | 0.934 | 0.784 | 0.477 |
| Deep Learning-AdaBoost | test | 0.593 | 0.577（0.451 - 0.704） | 0.361 | 0.778 | 0.565 | 0.603 | 0.441 | 0.532 |
| Deep Learning-MLP | training | 0.783 | 0.862（0.811 - 0.913） | 0.790 | 0.778 | 0.727 | 0.832 | 0.757 | 0.487 |
| Deep Learning-MLP | test | 0.691 | 0.712（0.597 - 0.828） | 0.750 | 0.644 | 0.628 | 0.763 | 0.684 | 0.450 |
| Combined-LR | training | 0.767 | 0.822（0.763 - 0.881） | 0.630 | 0.870 | 0.785 | 0.758 | 0.699 | 0.623 |
| Combined-LR | test | 0.778 | 0.823（0.732 - 0.915） | 0.889 | 0.689 | 0.696 | 0.886 | 0.780 | 0.480 |
| Combined-NaiveBayes | training | 0.762 | 0.804（0.741 - 0.868） | 0.765 | 0.759 | 0.705 | 0.812 | 0.734 | 0.254 |
| Combined-NaiveBayes | test | 0.741 | 0.802（0.705 - 0.898） | 0.917 | 0.600 | 0.647 | 0.900 | 0.759 | 0.217 |
| Combined-SVM | training | 0.820 | 0.852（0.796 - 0.909） | 0.741 | 0.880 | 0.822 | 0.819 | 0.779 | 0.593 |
| Combined-SVM | test | 0.753 | 0.804（0.709 - 0.899） | 0.972 | 0.578 | 0.648 | 0.963 | 0.778 | 0.330 |
| Combined-KNN | training | 0.778 | 0.867（0.819 - 0.916） | 0.654 | 0.870 | 0.791 | 0.770 | 0.716 | 0.600 |
| Combined-KNN | test | 0.704 | 0.798（0.706 - 0.890） | 0.583 | 0.800 | 0.700 | 0.706 | 0.636 | 0.600 |
| Combined-XGBoost | training | 0.868 | 0.918（0.876 - 0.960） | 0.802 | 0.917 | 0.878 | 0.861 | 0.839 | 0.519 |
| Combined-XGBoost | test | 0.679 | 0.697（0.582 - 0.811） | 0.639 | 0.711 | 0.639 | 0.711 | 0.639 | 0.508 |
| Combined-LightGBM | training | 0.820 | 0.868（0.814 - 0.922） | 0.778 | 0.852 | 0.797 | 0.836 | 0.787 | 0.510 |
| Combined-LightGBM | test | 0.605 | 0.706（0.592 - 0.819） | 0.806 | 0.444 | 0.537 | 0.741 | 0.644 | 0.458 |
| Combined-AdaBoost | training | 0.820 | 0.887（0.842 - 0.932） | 0.827 | 0.815 | 0.770 | 0.863 | 0.798 | 0.502 |
| Combined-AdaBoost | test | 0.679 | 0.741（0.633 - 0.849） | 0.806 | 0.578 | 0.604 | 0.788 | 0.690 | 0.468 |
| Combined-MLP | training | 0.841 | 0.909（0.868 - 0.950） | 0.852 | 0.833 | 0.793 | 0.882 | 0.821 | 0.470 |
| Combined-MLP | test | 0.765 | 0.846（0.763 - 0.928） | 0.944 | 0.622 | 0.667 | 0.933 | 0.782 | 0.335 |

Supplementary Table 3. Diagnostic performance for all models.


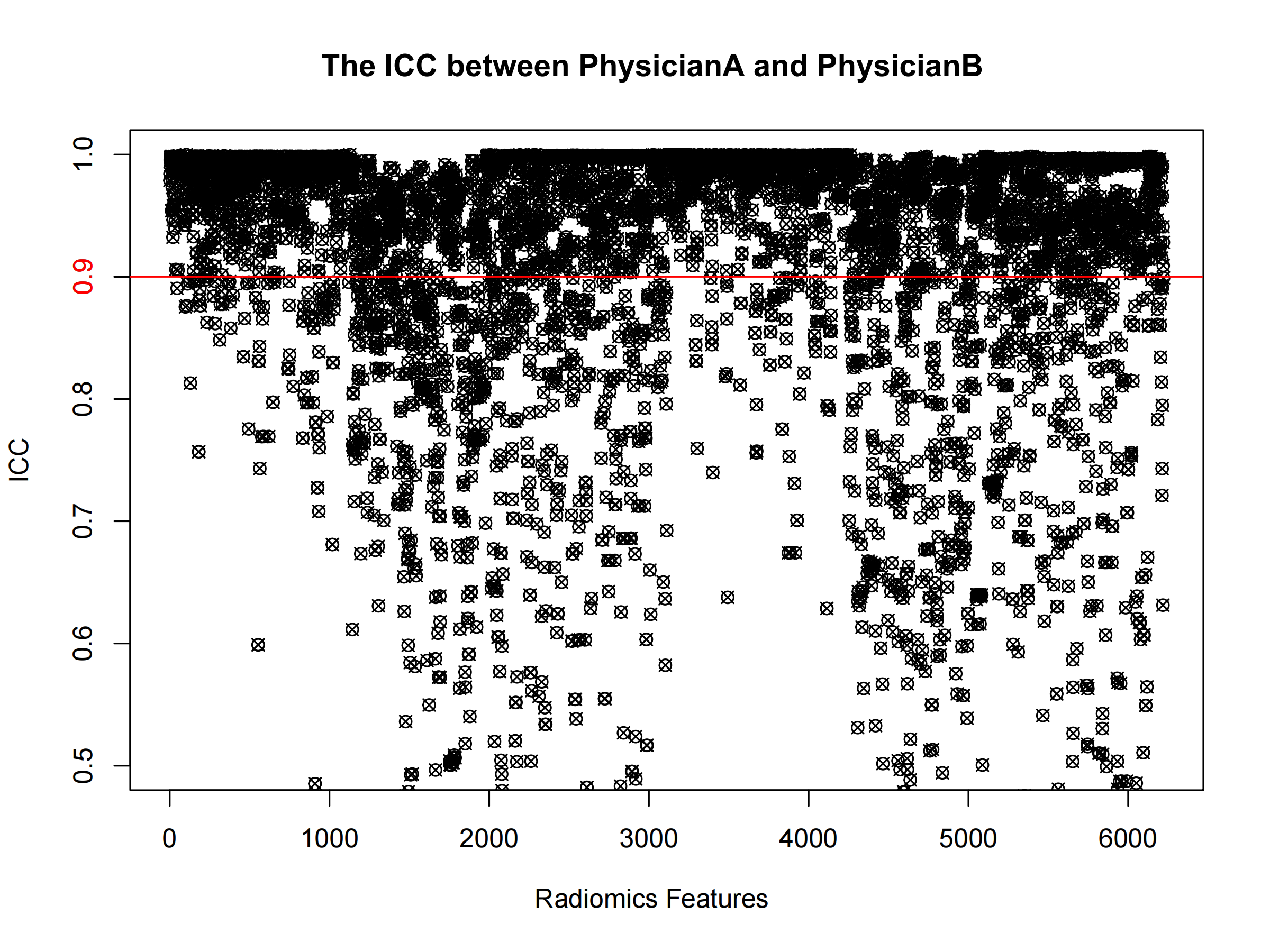


Supplementary Fig. 1. ICC index plot. Each point represents a radiomics feature. We retained the radiomics features with an ICC > 0.9 (above the red line).


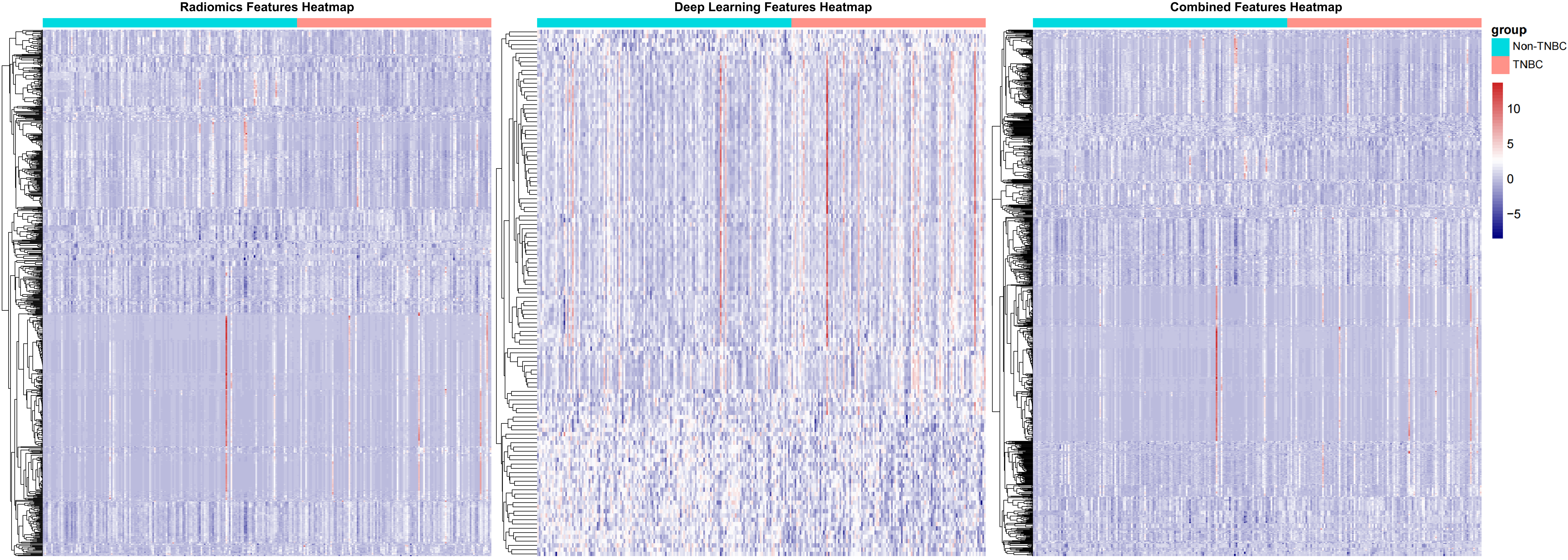


Supplementary Fig. 2. Heatmaps of feature clustering analysis. Features selected through univariate analysis were used for plotting. Red indicates high feature values, while blue indicates low values.


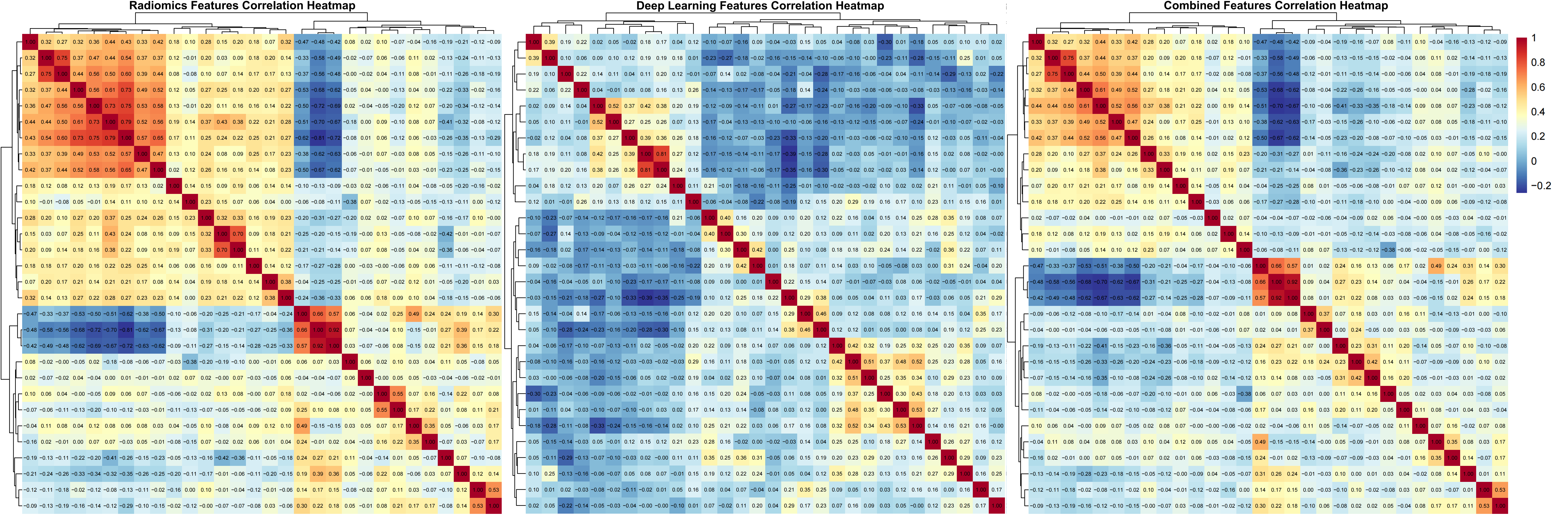


Supplementary Fig. 3. Heatmaps of feature correlations after Spearman correlation test and mRMR, showing all feature correlations < 0.9.


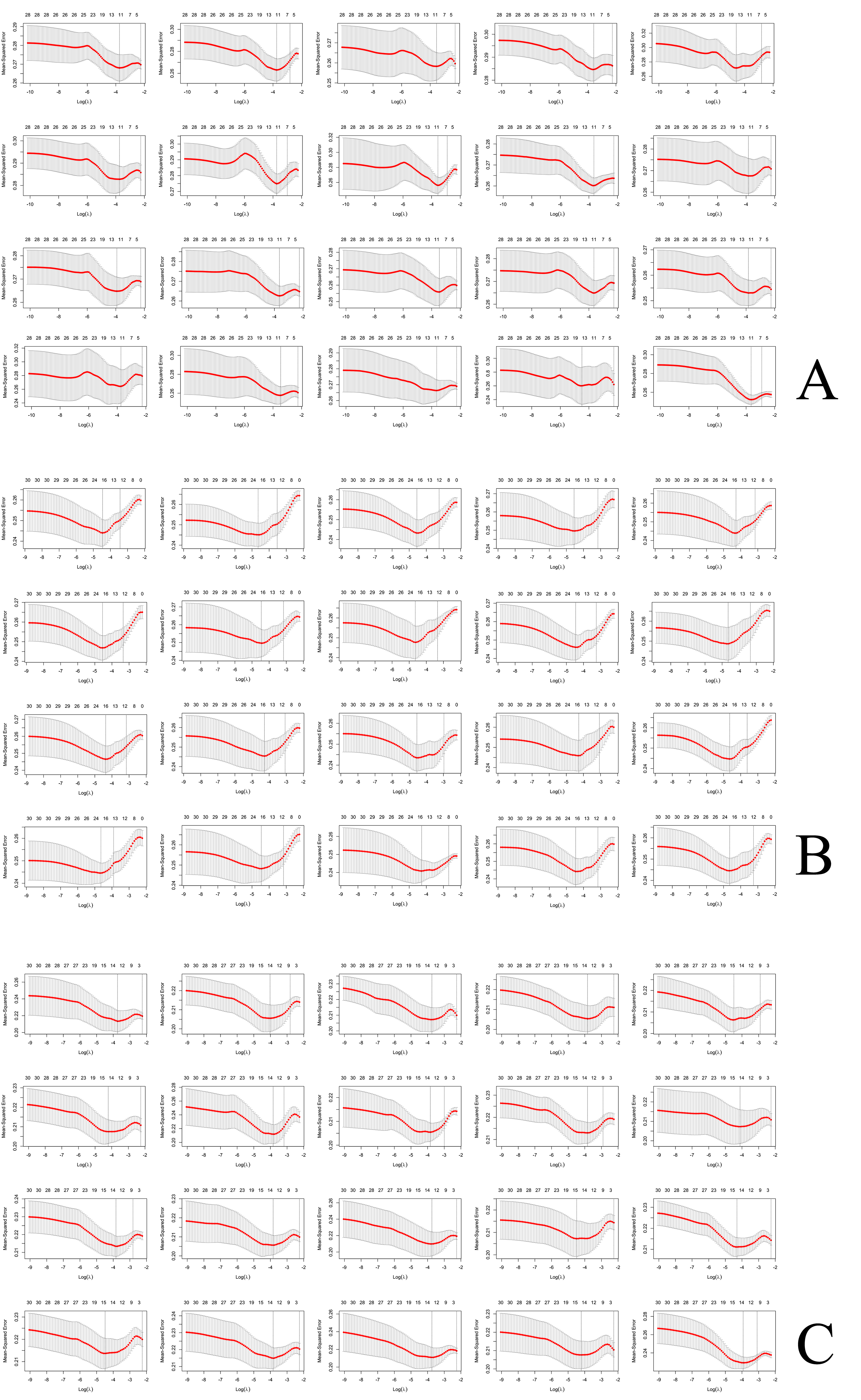


Supplementary Fig. 4. Repeated 10-Fold Cross-Validation with Different Random Seeds. A: Radiomics models. B: Deep learning models. C: Combined models.


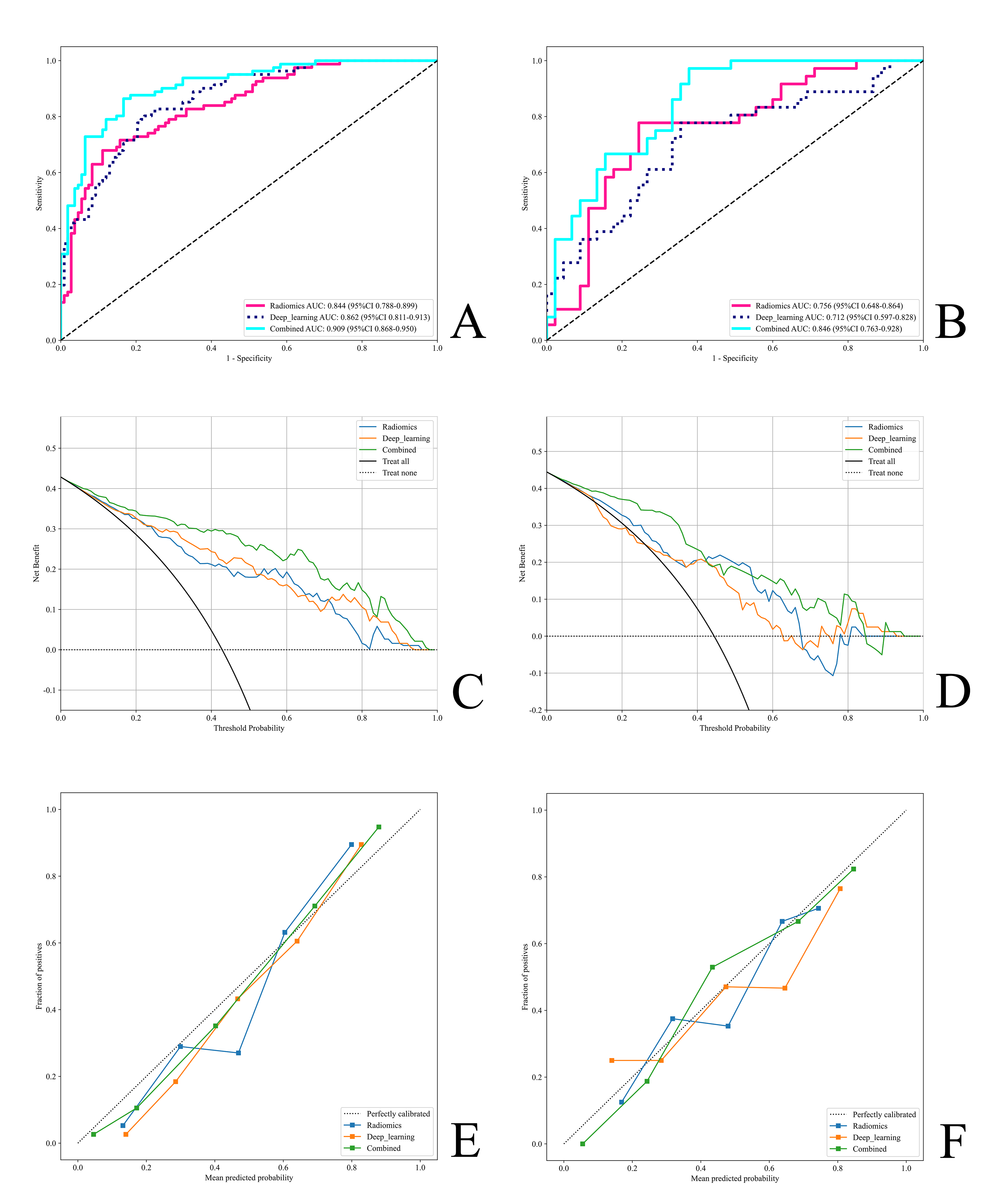


Supplementary Fig. 5. ROC, CAL, and DCA of the MLP model. A, B: ROC. C, D: CAL. E, F: DCA. A, C, E: Training set. B, D, F: Test set.


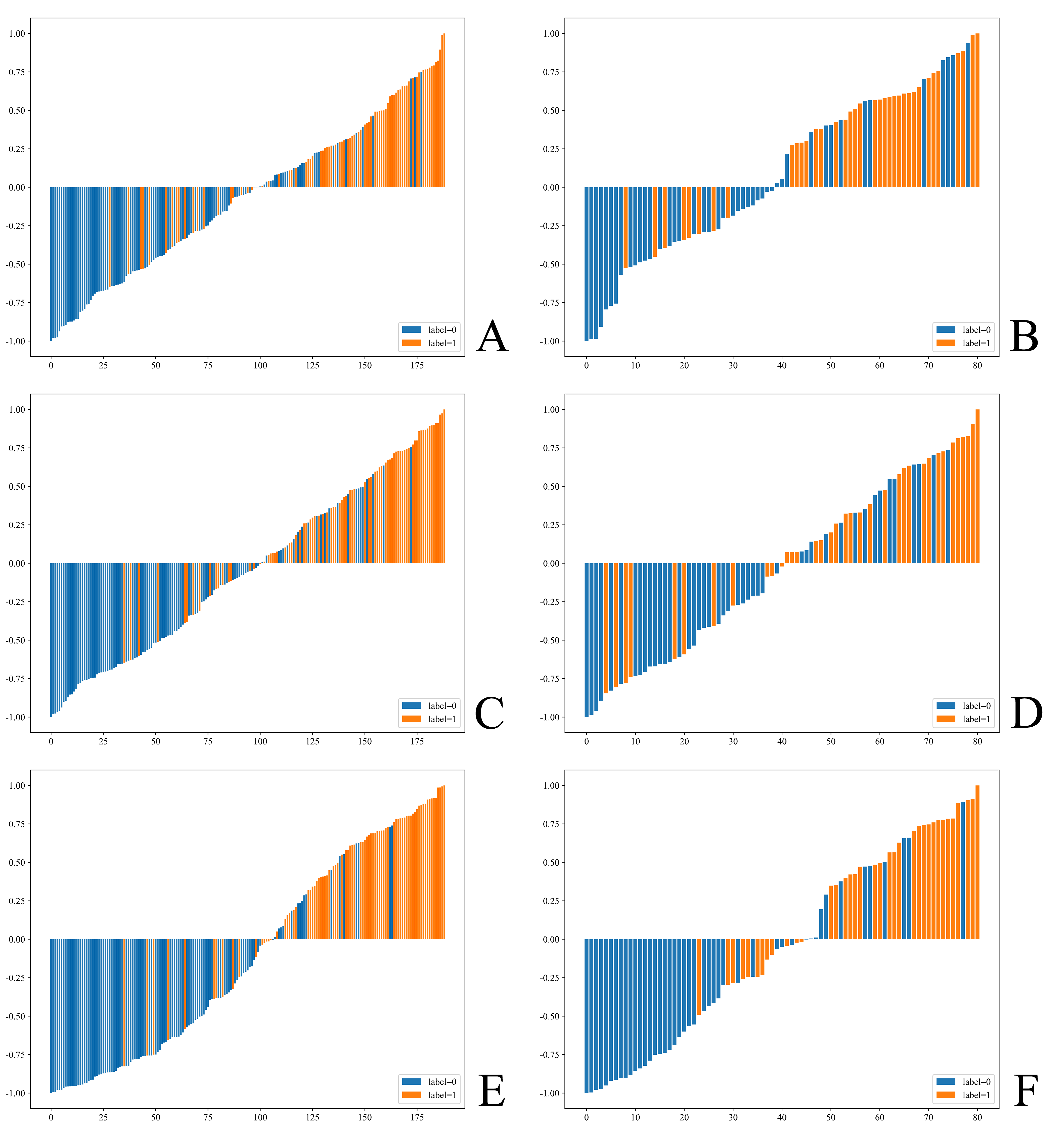


Supplementary Fig. 6. Waterfall plot of the MLP model. Blue columns (Label-0): Non-ALNM patients. Yellow columns (Label-1): ALNM patients. A, B: Radiomics models. C, D: Deep learning models. E, F: Combined models. A, C, E: Training set. B, D, F: Test set.


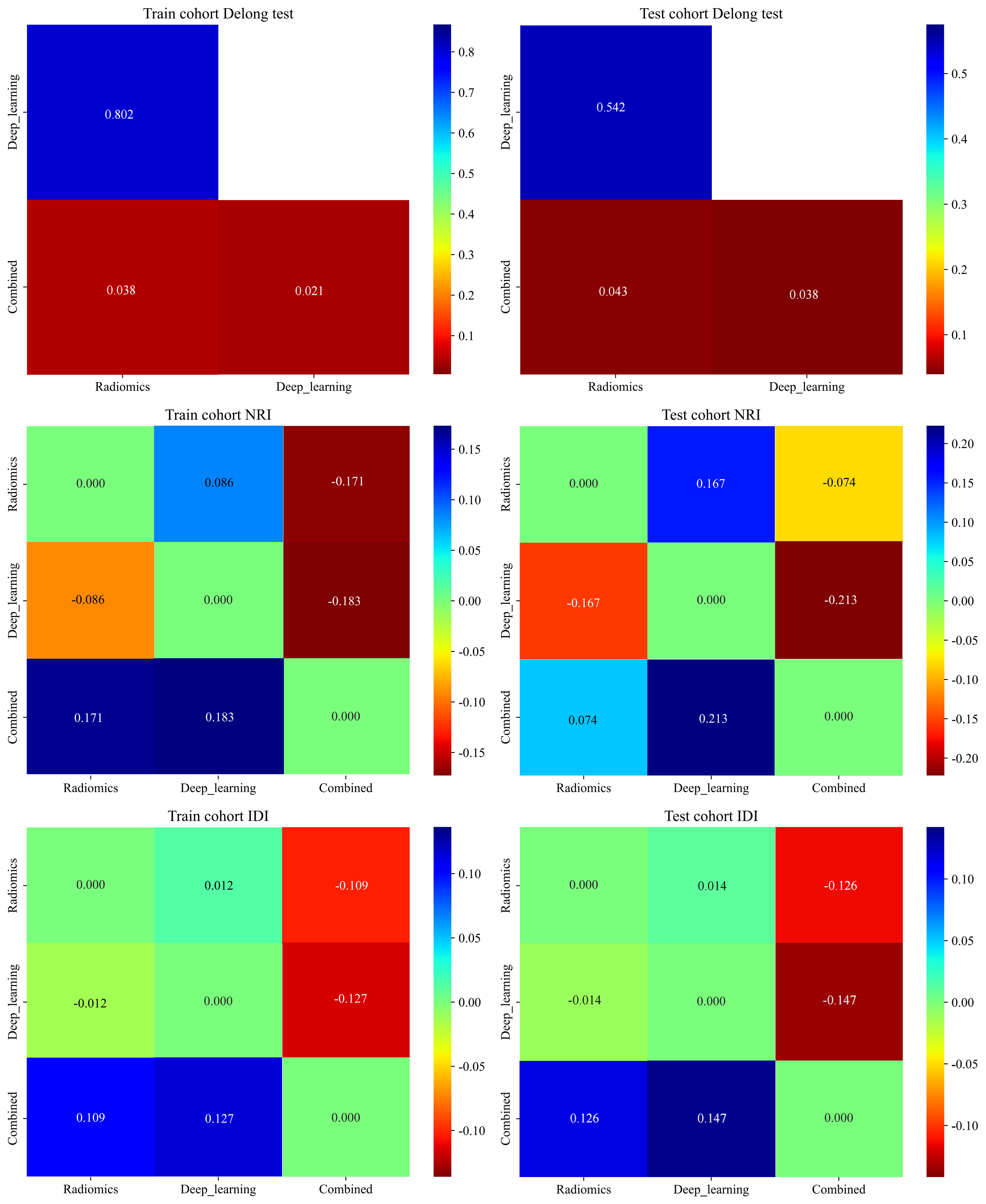


Supplementary Fig. 7. The results of the DeLong test, NRI, and IDI for the MLP model.
